# Caprine prion gene polymorphisms are associated with decreased incidence of classical scrapie in goat herds in the United Kingdom

**DOI:** 10.1186/1297-9716-42-110

**Published:** 2011-10-31

**Authors:** Wilfred Goldmann, Kelly Ryan, Paula Stewart, David Parnham, Rosa Xicohtencatl, Nora Fernandez, Ginny Saunders, Otto Windl, Lorenzo González, Alex Bossers, James Foster

**Affiliations:** 1 The Roslin Institute and Royal (Dick) School of Veterinary Studies, University of Edinburgh, Easter Bush, Midlothian, UK; 2 Facultad de Medicina Veterinaria y Zootecnia, Benemérita Universidad Autónoma de Puebla, Puebla, México; 3 Animal Health and Veterinary Laboratories Agency, Weybridge, New Haw, Addlestone, Surrey, UK; 4 Animal Health and Veterinary Laboratories Agency, Pentlands Science Park, Bush Loan, Penicuik, Midlothian, UK; 5 Central Veterinary Institute of Wageningen UR, Lelystad, The Netherlands

## Abstract

The application of genetic breeding programmes to eradicate transmissible spongiform encephalopathies in goats is an important aim for reasons of animal welfare as well as human food safety and food security. Based on the positive impact of *Prnp *genetics on sheep scrapie in Europe in the past decade, we have established caprine *Prnp *gene variation in more than 1100 goats from the United Kingdom and studied the association of *Prnp *alleles with disease phenotypes in 150 scrapie-positive goats. This investigation confirms the association of the Met142 encoding *Prnp *allele with increased resistance to preclinical and clinical scrapie. It reveals a novel association of the Ser127 encoding allele with a reduced probability to develop clinical signs of scrapie in goats that are already positive for the accumulation of disease-specific prion protein in brain or periphery. A United Kingdom survey of *Prnp *genotypes in eight common breeds revealed eleven alleles in over thirty genotypes. The Met142 encoding allele had a high overall mean allele frequency of 22.6%, whereas the Ser127 encoding allele frequency was considerably lower with 6.4%. In contrast, a well known resistance associated allele encoding Lys222 was found to be rare (0.9%) in this survey. The analysis of *Prnp *genotypes in Mexican Criollas goats revealed nine alleles, including a novel Phe to Leu substitution in codon 201, confirming that high genetic variability of *Prnp *can be found in scrapie-free populations. Our study implies that it should be feasible to lower scrapie prevalence in goat herds in the United Kingdom by genetic selection.

## Introduction

The goat population in the United Kingdom (UK) is small compared to other European countries and contributes only a minor fraction of the total livestock production. However, in contrast to sheep, natural cases of bovine spongiform encephalopathy (BSE) in goats have been reported in France and the UK [1,2] which highlights the need for more knowledge regarding the susceptibility of goats to transmissible spongiform encephalopathies (TSEs), a group of disorders also known as prion diseases [for review see [3,4]].

The number of goats in the UK is relatively constant with 85-90 thousand animals held at an average herd size below 20; there are less than 50 farms keeping 200 or more goats. Just over 63% of these are milk-producing Saanen, Toggenburg, British Alpine or Anglo- Nubian goats. Around 17% are meat-producing goats, with a high proportion of the Boer breed, the remaining animals breeding stock or companion animals [5]. Feral goats are found in many rocky areas of the British isles, they are a mixture of various breeds of wild and domestic goats [6].

TSEs are fatal neurodegenerative diseases known to affect many mammalian species, including humans. TSEs in animals include scrapie in sheep and goats, bovine spongiform encephalopathy (BSE) in cattle, and chronic wasting disease (CWD) in cervids. Scrapie is not regarded as a human health risk, whereas BSE has been transmitted to humans in the form of variant Creutzfeldt-Jakob disease [7]. A common feature of TSEs is the accumulation, mainly in the brain, of disease-associated prion protein (PrP^d^), an aberrant isoform of the normal, host-encoded cellular prion protein (PrP^C^). This accumulation is thought to result from the conversion of PrP^C ^to PrP^d^. PrP^d ^is considered by many to be the infectious agent of TSEs [8]. Goat scrapie has been reported in 9 out of the 27 European Union member states and cases have also been seen in the USA and Canada [reviewed in [9]]. Active and passive surveillance in the UK has resulted in the detection of several goat scrapie outbreaks [10] which form part of the study presented here.

Caprine PrP^C ^is encoded by a single gene *(Prnp) *for which at least twenty-eight amino acid substitutions exist in goat breeds throughout the world [9]. This is very similar to the genetic variability seen in domestic sheep, for which *Prnp *genetics and TSE association have been developed much further. Of more than 40 amino acid substitutions recorded for ovine PrP^C^, at least ten have been shown to exhibit significant modulating effects on scrapie. Of these only one increases susceptibility for classical scrapie (codon 136) and two are proven to enhance susceptibility to atypical scrapie (codons 141 and 154) [11-13]. Based on this information large scale breeding programs have been established in several EU countries aimed at eradicating scrapie from national flocks by breeding for genetic resistance, at the same time dealing with the theoretical risk of BSE in sheep. The question whether equivalent genetic breeding programmes could be possible for UK goats is addressed in this study.

Association studies between *Prnp *genetics and susceptibility to scrapie in goats are so far limited and often restricted in their statistical significance. Nonetheless, some caprine *Prnp *alleles have been implicated as providing increased resistance to disease development relative to wild-type alleles. Importantly, novel polymorphisms that increase susceptibility have not yet been discovered, although goats share with sheep the association of codon 154 histidine (H154) with increased susceptibility to atypical scrapie [13]. The PrP variant encoding lysine in codon 222 (K222) has been associated with highly significant protection from scrapie in Italian and French studies [14,15], whereas the codon 146 serine (S146) and 146 aspartic acid (D146) variants have been similarly associated with scrapie resistance in Cypriot herds [16,17]. Modulation of classical scrapie by polymorphisms in codons 143, 154 and 211 has been suggested by studies from France, and Greece [14,18,19]. The association of 142 methionine (M142) with lengthening of the incubation periods after experimental scrapie or BSE challenge was shown by us some years ago [20] whereas recently the M142 allele was shown to modulate genetic susceptibility in a single high incidence herd in the UK [21,22].

This study provides further evidence that the M142 allele has scrapie protective characteristics and that it is present at high frequency in dairy breeds in the UK. We also show that there are important differences between the *Prnp *allele frequencies of the UK and other countries, and that high genetic variability of *Prnp *can be found in small, scrapie-free populations.

## Materials and methods

Goat blood samples from British herds were collected through veterinary surgeries and the Veterinary Services. The male to female ratio was about 1:9. The mean age of the animals was 45 months (standard deviation 30 months, range: 2 to 156 months). Blood samples were collected from a total of 22 UK holdings representing all commercially used breeds in the UK. A large proportion of the sampled animals were crossbreeds. Each herd sampling represented 10-50% of the total herd size, with the exception of herd B, for which all animals were collected. Feral goat tissue samples were collected as part of an annual cull in the north west of Scotland in 2008 [6]. Blood samples from Criollas goats were obtained from yearling and adult goats from grazing flocks in ten villages located in the South of Puebla and three villages in the North-West of Oaxaca State, Mexico.

**Figure 1:**
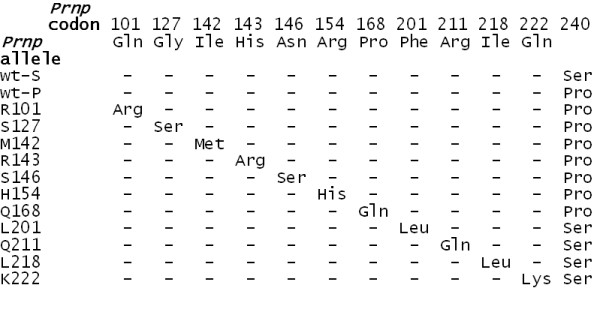
**Caprine *Prnp *alleles as defined by twelve codon positions**.

DNA was extracted from blood or tissue using the Qiagen DNeasy blood & tissue kit^® ^(Qiagen, Crawley, UK). PCR was performed using primers PS-141d GGAATGTGAAGAACATTTATGACCTAGAAT and PS+109u CAAGAGAGAAGCAAGAAATGAGACA. PCR conditions were 95°C for 5 min followed by 40 cycles of 95°C 30 s, 62°C 30 s and 72°C 1 min. PCR fragments were directly sequenced using an Applied Biosystems 3130 Genetic Analyzer with forward and reverse primers P61+ AACCAACATGAAGCATGTGG and 961 GGTGAAGTTCTCCCCCTTGGT with BigDye^® ^reagents (Life Technologies, Paisley, UK) as recommended by the manufacturer. The data was analysed manually using ABI sequence scanner V1.0. The full open reading frame was read and all variation was recorded. Alleles and genotypes are based on codons 101, 127, 142, 143, 146, 154, 211, 218, 222 and 240 (all other positions being equal) and described by the name shown in Figure 1. As discussed later alleles with proline or serine at codon 240 are both considered to be wildtype. Genotypes are shown as eg. IM_142 _or GG_127_. Differences in allele and genotype frequencies were tested for significance using Fisher's exact test.

Material from scrapie-affected goats available to this study (*n *= 150) was collected by the UK Veterinary Services from three herds over a period of approximately three years following passive (here: clinical cases) and active surveillance (here: fallen stock or as part of research activity). They were confirmed by immunohistochemical and/or biochemical diagnosis for presence of PrP^d ^(scrapie-affected) or absence of PrP^d ^(scrapie-free) either at the AHVLA, Weybridge which is also the European Union Reference Laboratory for TSEs or at AHVLA, Bush Loan as described by González et al. [21]. Animals that were not tested for PrP^d ^but were clinically negative at time of cull are described as clinically negative.

## Results

### Association of *Prnp *polymorphisms with scrapie

We compared the allele frequencies in the four scrapie affected herds (A, B, E, J) with dairy breed herds that had no confirmed scrapie cases (see Tables 1). There was no significant difference in the allele frequencies between the two groups, with the exception of herd B which had a very high frequency of the S127 allele (25%).

**Table 1:** Comparison of *Prnp *allele frequencies [%] between scrapie-affected and healthy herds.

		Scrapie Herds (A, B, E, J)	Herds without scrapie cases (C, D, F, G, H, I, X)
**Breed category**		Dairy	Dairy

**Breed**		S, T, A, N	S, T, A, N

**Number of animals**		699	235

**Allele frequencies [%]**	wt-S	14.5	15.8
	
	wt-P	45.5	51.9
	
	S127	10.2	2.3
	
	S127*	4.5 (A, E, J only)	2.3
	
	M142	26.9	25.5
	
	Q211	2.2	2.6
	
	K222	0.7	0.6

For abbreviations see Table 3 and Figure 1.

*S127 allele frequency after removal of herd B data.

The 150 scrapie cases available for genetic analysis came from herds A (*n *= 131), B (*n *= 16), and E (*n *= 3), none were collected from herd J. They can be divided into group-1 consisting of 78 animals collected as fallen stock or clinical positive animals A (*n *= 66), B (*n *= 9) and E (*n *= 3) and group-2 consisting of 72 pre-clinical PrP^d^-positive animals removed for research purposes during the cull of herds A (*n *= 65) and B (*n *= 7). The records for group-1 are not sufficiently detailed to identify unambiguously all clinically positive animals individually but it is known that a large number of them showed some signs of disease before they were culled.

The genetic analysis of the *Prnp *gene in group-1 goats revealed amino acid polymorphisms in codons 127, 142 and 240. When the combined S127 allele frequency for herds A and B are compared between scrapie-affected and clinically-negative animals a statistically highly significant difference (*p *= 1.7 × 10^-6^) was observed (1.4% versus 15.1%), which suggests that S127 protects from the appearance of clinical disease altogether or prolongs the incubation period. Herd E was not included in these calculations as the number of scrapie animals was very small. We considered that the inclusion of the M142 allele, which is associated with scrapie resistance [[14,20] and below] and the relative high frequency of S127 in the B herd may have skewed the disease association analysis. The frequencies of S127 were therefore also calculated after removing all M142 alleles resulting again in significance for herd A (0.8%: 8%, *p *= 0.0027), for herd B (5.6%: 33.3%, *p *= 0.016) and for A+B combined (1.4%: 19.5%, *p *= 2.5 × 10^-9^). The same disease association (*p *= 2.6 × 10^-11^) is found for GS_127 _versus GG_127 _genotypes in the combined herd analysis (3%: 44.6%; no M142 carriers), while the frequency of SS_127 _genotypes was too low to assess disease association (Table 2).

**Table 2:** *Prnp *genotype frequencies [%] of scrapie cases and control goats.

Herd Genotype	A Gp1 Scr+	A Gp2 Scr+	B Gp1 Scr+	B Gp2 Scr+	A Gp1/2 Scr+	B Gp1/2 Scr+	E Scr+	B Scr-	A^&^ Scr-	A^$^ cl. Scr-	E* cl. Scr-
**wt-S/wt-S**	6	3	0	0	4.6	0	0	0	2.3	6.6	0

**wt-P/wt-S**	34.8	12.3	11.1	0	23.7	6.3	66.7	1.3	4.6	21.7	12

**wt-P/wt-P**	43.9	21.5	77.8	57.1	32.8	68.7	33.3	24.6	12.5	21.2	22

**wt-P/S127**	1.5	4.6	11.1	42.9	3.1	25	0	28.3	8.6	6	3.3

**wt-S/S127**	0	7.7	0	0	3.9	0	0	1.9	0.8	2	0

**S127/S127**	0	0	0	0	0	0	0	5	0	0	0

**S127/M142**	0	1.6	0	0	0.8	0	0	11.9	2.3	3	3.3

**wt-P/M142**	10.6	29.3	0	0	19.7	0	0	20.1	30.5	19.7	31.9

**wt-S/M142**	1.6	15.4	0	0	8.4	0	0	0.6	16.4	7	7.7

**M142/M142**	0	3	0	0	1.5	0	0	5	17.2	7.6	9.9

**R101 carrier**	0	0	0	0	0	0	0	1.2	0	0	0

**Q211 carrier**	0	0	0	0	0	0	0	0	3.2	2.5	3.4

**K222 carrier**	0	0	0	0	0	0	0	0	0.8	1	5.5

**Partial^**	1.6	1.6	0	0	1.6	0	0	0	0.8	1.6	1.1

**Total (n)**	**66**	**65**	**9**	**7**	**131**	**16**	**3**	**159**	**128**	**198**	**91**

Legend: For allele abbreviation see Figure 1. Scr = scrapie; cl = clinical; for definition of group-1 (Gp1) and group-2 (Gp2) see main text, in short: group-1 clinical positive and fallen stock goats, group-2 pre-clinical, PrP^d ^positive goats; Gp1/2 = Gp1+Gp2; ^partial here means one allele in heterozygote could not be unambiguously identified for all positions; ^&^scrapie-free goats, clinically and PrP^d ^negative; ^$^clinically negative goats, randomly selected goats of which approx. 1/3 were tested and found negative for PrP^d^; *randomly selected clinically negative herd-mates, not tested for PrP^d^.

There was no significant association of S127 with disease in herds A and B when pre-clinical PrP^d^-positive goats (group 2) were compared to scrapie-free goats (Table 2) confirming that development of clinical disease and not the susceptibility to infection is associated with this PrP allele. Age information was missing for the only clinical positive GS_127_-II_142 _heterozygote goat from herd B, but from the mean age of all other GS_127_-II_142 _genotypes we conclude that there was no significant difference between scrapie-affected and scrapie-free goats for both herds. Analysis of herd A in this genotype group showed that the age of the clinical scrapie goat was 108 months, the eight group-2 heterozygotes had a mean age of 92 months (SD ± 17) and the scrapie-free goats had a mean age of 85.3 months (SD ± 25), with 50% being older than 100 months.

An association of codon 142 with scrapie susceptibility has previously been presented for herd A (group-2 animals only) by González et al., with the result that the probability of scrapie infection of II_142 _genotypes was significantly higher than for M142 carriers (*p *< 0.001) [21,22]. Here we extended the genetic analysis of this codon to all animals from herds A and B. When we compared the allele frequencies in scrapie-affected goats from group-1 in herd A and B with clinically negative goats the association of M142 with resistance was confirmed (*p *= 2.8 × 10^-6 ^and *p *= 0.03, respectively). Following the observation that the S127 allele may also modify disease we recalculated significance for the M142 allele with exclusion of the S127 allele which resulted in *p *= 8.8 × 10^-7 ^for herd A and *p *= 0.0044 for herd B.

Genotype frequencies for the scrapie-affected and healthy herd-mates are presented in Table 2. The nine B goats from group-1 were all genotype II_142 _whereas in herd A scrapie-affected goats could be divided into 87% genotype II_142 _and 13% genotype IM_142_. The frequency of IM_142 _combined in group-1 scrapie goats is 11%, which is significantly lower than the frequency of 35% for IM_142 _in healthy controls (*p *= 3.8 × 10^-5^), confirming the survival advantage of IM_142 _genotypes. Increased survival is also associated with the IM_142 _genotype in herd B (*p *= 0.007) when all scrapie animals are compared with scrapie-free goats. The low frequency of MM_142 _genotypes only allows statistical analysis of association for combined groups 1 and 2 from both herds. The MM_142 _genotype is then significantly (*p *< 0.005) associated with decreased incidence of scrapie as would be expected from the IM_142 _heterozygotes. The codon 240 polymorphism was neither associated with susceptibility of positive IM_142 _goats [14] nor with susceptibility in II_142 _genotypes. The frequencies of the Q211 and K222 alleles were too low to test for association, but it should be noted that none of the 150 scrapie cases carried either the Q211 or the K222 allele.

### PrP coding region variation in UK population

A large number of caprine *Prnp *gene polymorphisms have already been described but estimates of allele and genotype frequencies for the UK goat population have not been ascertained. In this UK study about 1200 animals have been genotyped for the full ORF of *Prnp*. Ten polymorphisms (codons 101, 127, 142, 143, 146, 154, 211, 218, 222, 240) lead to amino acid substitutions as shown in Figure 1. They all have been described before in goats [9,23,24] although Q101R, N146S and I218L were observed in British goats for the first time. The nine polymorphisms from codons 101 to 222 are all mutually exclusive, whereas proline or serine encoded in position 240 are found in various combinations with the other amino acids (Figure 1).

We determined the allele frequencies for the three main breed categories, which are dairy (D), fibre (F) and meat (M) goats. The archetypal and wildtype alleles wt-S, expressing serine at 240 and its variant wt-P, expressing proline at 240 are the most common alleles with frequencies between 56% and 89%. Whereas wt-P is the majority wildtype allele in dairy and fibre breeds (76.2% and 62.7%, respectively), the reverse is found in meat breeds with wt-S dominating with 70.5% (Tables 3 and 4).

**Table 3:** Percentage frequencies of polymorphisms in the caprine *Prnp *open reading frame in UK farms.

Herd	A$	B$	C	D	E$	F	G	H	I	X	J$	K	L	M	P	Q	R	S	T
**Breed Category**	**Dairy**										**Dairy** **Meat**	**Fiber**		**Fiber** **Meat**	**Meat**			**Other**	

**Breed**	**S, T, A, N**	**S, A**	**S, T**	**S, T**	**S**	**S**	**S, A**	**S, T, A, N**	**S**	**S, T**	**S, B**	**Ag**	**Ag**	**Ag, C, B**	**B**	**B**	**B**	**GG**	**F**

**Number**	323	188	20	39	91	35	23	29	48	41	97	29	22	35	103	21	29	12	10

**wt-P & wt-S**	62.9	50.8	50	60.2	59.3	57.1	76.2	96.6	61.5	75.7	63.4	96.6	95.5	75.7	52.4	57.1	69	16.7	100

**R101**	-	0.5	**-**	-	-	-	-	-	-	-	-	-	-	-	-	-	-	-	-

**S127**	5.3	25.5	10	-	3.3	4.3	4.3	-	-	2.4	3.1	-	-	-	-	2.4	-	-	-

**M142**	29.1	23.2	32.5	37.2	33	27.1	15.2	-	37.5	19.5	20.8	1.7	-	1.4	19.4	2.4	-	-	-

**R143**	-	-	-	-	-	-	-	3.4	-	-	-	1.7	4.5	8.6	2.9	2.4	12	-	-

**S146**	-	-	7.5	-	-	-	-	-	-	-	4.1	-	-	4.3	23.3	33.3	19	-	-

**H154**	-	-	-	-	-	-	-	-	-	-	-	-	-	1.4	0.5	-	-	-	-

**Q211**	1.9	-	-	1.3	3.3	8.6	4.3	-	1	2.4	6.8	-	-	-	-	2.4	-	50	-

**L218**	-	-	**-**	-	-	-	-	-	-	-	-	-	-	8.6	1.5	-	-	-	-

**K222**	0.8	-	-	1.3	1.1	2.9	-	-	-	-	1.6	-	-	-	-	-	-	33.3	-

Breeds: A = Alpine, Ag = Angora, B = Boer, C = Cashmere, GG = Golden Guernsey, N = Anglo-Nubian, S = Saanen, T = Toggenburg, F = Feral; number = number of animals, for calculation of allele frequency this is doubled to get number of chromosomes. For codon 101 only 2350 chromosomes were recorded and for codon 127 only 2362. X represents five farms each ≤ 12 animals. ^$^scrapie-affected herds.

**Table 4:** UK mean *Prnp *polymorphism frequencies [%] and comparison with frequencies from other countries.

Country	UK				Mexico	France^1^	Italy^2^
**Breed Category**	**Dairy**	**Meat**	**Fiber**	**ALL**	**Meat**	**Dairy**	**Dairy**

**Number**	932	157	84	1195	166	404	478

**wt-P & wt-S**	61.8	56.1	89.2	62.7	62	66.4	67.8

**R101**	0.1	-	-	0.1	-	-	-

**S127**	8.2	0.3	-	6.4	-	5.8	1.8

**M142**	26.6	13.2	1.2	22.6	14.5	6.1	8.4

**R143**	0.1	4.5	5.4	1.0	0.3	-	1.3

**S146**	0.2	24.5	-	3.6	5.4	-	-

**H154**	-	0.3	0.6	0.1	3.3	3.2	5.3

**Q168**	-	-	-	-	3	-	0.4

**L201**	-	-	-	-	1.5	-	-

**Q211**	2.3	0.3	-	2.3	10	12.2	7.4

**L218**	-	0.7	3.6	0.4	-	-	-

**K222**	0.7	-	-	0.9	-	6.3	7.6

References ^1^[14]^2^[26]. See also table 3.

The 934 animals belonging to the dairy breeds were from 15 farms and represented all four common dairy breeds. More than a third of *Prnp *alleles (38.2%) showed one of seven amino acid substitution, the most common change being M142 with an allele frequency for all dairy goats of 26.6%. Two further alleles with frequencies above one percent were S127 and Q211. Minor alleles with less than 1% were R101, R143, S146 and K222 (Table 3). However, a significant increase in the frequency of the minor alleles could occasionally be observed, eg. in herd F the frequency of K222 was four-times higher than the mean frequency for all dairy goats.

The 84 Angora and Cashmere goats contributing to the analysis of the hair (fibre) breeds showed in contrast to the previous group a very low frequency of the M142 allele. They had however the highest frequency of the R143 allele amongst all breeds (5.4%) and the alleles H154 (0.6%) and L218 (3.6%) were only seen in this group. There were no carriers of the polymorphisms in codons 127, 211 and 222.

The meat category was represented with 157 animals from the Boer breed. The most common substitution was S146 with 24.5% allele frequency followed by M142 with 13.2%. The frequencies of the other alleles are below 1% with the exception of R143 at 4.5%. No carriers of the K222 allele were found.

Ten feral goats from Scotland (K6) were analysed and they were the only group that was 100% wildtype, but both alleles, wt-S and wt-P, were found at frequencies of 25% and 75%, respectively. Twelve purebred Golden Guernsey goats from one farm (S) had a very low wt-allele frequency (16.7%) with all animals either carrying the K222 allele (33.3%) or the Q211 allele (50%).

We then analysed the genotype frequencies for the 1200 animals. The three most common genotypes making up almost 60% of all animals were wt-P/wt-P (21%), wt-P/M142 (20.5%) and wt-P/wt-S (16%). When the common polymorphism at codon 240 is disregarded the number of genotypes was 22, but half of those had frequencies of less than 1%. When all wt/wt homozygotes are removed from the frequency calculations, M142 carriers are 61% followed by S127 (15%), S146 (11%) and Q211 (8%). Rare genotypes containing R143 and K222 had frequencies of only 3.5% and 2.9%, respectively.

The dairy breed category was not very different from the all-breed average with the highest frequencies for wt/wt of 38% and wt/M142 of 32.7%. Seven genotypes were less frequent than 1%. Q211-and K222-carrying genotypes were slightly more infrequent than in the overall assessment with 4.4% and 1.4%, respectively. The meat breed group showed a significant increase of S146 carriers (44%) which is not linked to a reduction in the M142 carriers (21%) when compared to the results from all breeds.

### PrP polymorphisms in Mexican goats

It has been argued for sheep that PrP alleles are maintained in populations through balancing selection leading to the speculation that scrapie may provide the selection pressure [25]. We have provided evidence that the UK goat population has a high number of PrP variants in the gene-pool, similar to populations in France and Italy (Table 4). To compare these data with goats of European descent but independently propagated for centuries in a country officially free of scrapie, goats (*n *= 166) were genotyped from different areas of Central Mexico. As shown in Table 4 and Figure 1, eight polymorphisms were detected, one of them was novel, a substitution of phenylalanine (F) with leucine (L) in codon 201 due to a c → g transversion in the third position. This leucine change was found in linkage with S240. Another polymorphism (P168Q) has not been seen in UK goats or outside Europe, but has been described for Italian, Greek and Cypriot goats.

Comparable allele frequencies to the UK and other European populations were found in the Criollas goats, with M142 and S146 at frequencies of 14.5% and 5.4%, respectively. The frequency of the Q211 allele of 10% was surprisingly high as the frequency in the UK Boer goats was almost two orders of magnitude lower. The two most common genotypes making up almost 56% of all animals were wt/wt (38%, any combination in codon 240) and M142/wt-P + M142/wt-S (18%). Eight genotypes had frequencies of less than 1%. When all wildtype homozygotes are removed from the frequency calculations, M142 carriers were most common with 43% followed by the Q211 (30%) and S146 (27%) carriers. Excluding codon 240, 57% of the animals were heterozygous genotypes.

## Discussion

This study provides new evidence in UK goats for an association of *Prnp *gene polymorphisms with low disease incidence and probably with partial resistance to classical scrapie. In contrast there is no indication for gene variants associated with increased susceptibility to classical scrapie, such as the one found for codon 136 valine polymorphism of ovine *Prnp *[12]. Our genetic analysis of classical scrapie cases from dairy goats in two UK herds collected within a period of about 3 years reveals for the first time an association of codon 127 serine with a decreased probability to develop clinical scrapie, but not with susceptibility to infection and accumulation of PrP^d^. The development of clinical signs is a measure of incubation period length and defines the age-of-onset in natural scrapie cases, our data imply that S127 is a modulator of pathogenesis, similar to other Prnp alleles in ruminants, eg. H154 in sheep or 132L in deer [12]. Whether goats with S127 containing genotypes are lifelong subclinical carriers of infectivity or have a very extended incubation period remains to be investigated.

A similar association was observed with the codon 142 methionine carriers, where the frequency of clinical positive M142 carriers is lower than compared to the frequency of PrP^d^-positive, preclinical M142 carriers. But for both, clinical and preclinical, the frequency is significantly lower than for II_142 _genotypes. There was no susceptibility difference between M142/wt-S and M142/wt-P genotypes as has been suggested in studies by Barillet et al. [14].

Reported classical scrapie cases have been absent from the UK goat population for more than 10 years before these four affected herds emerged [21]. Little is known about the origin of these scrapie outbreaks and they represent for the time being the only UK herds to study *Prnp *disease association. Our data are therefore subject to the relative small number of cases and confirmation of the described genetics, particularly for S127 will depend on further outbreaks or experimental challenges. However, our findings regarding the M142 allele are in agreement with previous publications [14,20,21].

Of the ten *Prnp *coding region polymorphisms that were observed in our herds, three (Q101R, R154H and N146S) are described for the first time in the UK goat population. There are important differences in the frequencies of these polymorphisms between the various breeds. The M142 allele is less prominent in the meat and fibre breeds; to our best knowledge these breeds have never reported scrapie cases in the UK. However the Boer goats showed high frequency of the polymorphism N146S which has been shown in the Cypriot Damascus breed to increase scrapie resistance [16,17]. This polymorphism was also reported in Boer goat in Chinese populations, in which the S146 allele is the more frequent (57.4%). Only the Cashmere goats carried the I218L polymorphism, which is consistent with data from Chinese studies which showed this polymorphism in Liaoning Cashmere and Beijing Native goats at frequencies of 13.3% and 62.5%, respectively [23]. The *Prnp *analysis suggest that the UK meat and fibre breeds are closer to each other than they are to dairy breeds, which is supported by other breed characteristics. The H154 allele, which is proposed to increase the susceptibility to atypical scrapie, was not observed in the dairy breeds, which make up the majority of UK goats. In contrast, the S127 allele was found almost exclusively in dairy goats where it showed high variation between herds (2.5%-25%), although the mean was similar to populations in some other countries.

The frequencies of some of the *Prnp *alleles in the UK compared to some other countries are quite different (Table 4) with the exception of the wildtype alleles. This is an important issue when breeding strategies are to be considered, which are dependent on allele selection and potential QTL linkage. Almost all UK dairy goat herds had a high percentage (≥ 20%) of the M142 allele, which is three times higher than for herds from France and Italy [14,26]. The fact that this allele is found at this high frequency in the UK goat population is surprising as it could suggest a selective advantage. However, it would be very speculative to assume that this resistance-related allele is present and maintained at that level due to previous or current scrapie epidemics. It should be noted here that before *Prnp *genotype-based breeding was introduced into the UK sheep population the resistance related R171 allele was also already frequent [27], which may have been due to balancing selection [25]. The association of the M142 allele with partial resistance and its high frequency would make it a candidate for breeding programmes to reduce scrapie prevalence in the short term.

A marked difference was found in polymorphisms R211Q and Q222K which were four times and ten times less common, respectively, in British herds than in French and Italian herds. Because of the very high resistance to TSEs conferred by the K222 allele, it presents a good candidate allele to select for in genetic scrapie eradication programmes. Our data show that 33% of dairy herds contain at least one K222 carrier, whereas none were found in the herds of the meat and fibre breeds. With a K222 allele frequency of less than 1% on average, natural breeding to high levels will remain a long term aim rather than a immediate solution for the UK. However dairy goats in Western European countries are less diversified into breeds than sheep and the utilization of European breeding stock with the K222 allele through artificial insemination may be a viable option.

It has been reported that human and sheep *Prnp *are under balancing selection [25,28], which may or may not be related to TSEs. A consequence of this would be the maintenance of a larger number of *Prnp *variants in the population than under purifying selection, which has been suggested to constrain bovine *Prnp *variability [29] and may underlie wild populations such as chamois and deer [30,31]. It appears that domestic goat populations maintain a number of *Prnp *polymorphisms as might be expected from balancing selection and the majority of these polymorphisms is associated with a degree of protection against TSEs compared to the wildtype. We were interested to see if a goat population from a region which had never any confirmed scrapie outbreaks would show equally diverse *Prnp *genetics as the UK or other scrapie affected countries. We selected herds of Mexican Criollas goats, a breed of European linage that was introduced several hundred years ago into the Americas. These goats would most likely not have been under TSE-related selection. The fact that we found five polymorphisms at frequencies ≥ 3% and a novel F201L polymorphism in fewer than 200 animals leads us to argue that TSE epidemics are not the exclusive reason for high genetic variability of the *Prnp *in livestock species such as sheep and goats. Interestingly, the K222 allele most closely associated with high scrapie resistance in French flocks [14] was not detected in Mexican Criollas goats, whereas the S146 allele -associated with scrapie resistance in Cypriot flocks [16,17]-was present at 5% level.

This study confirmed that the UK goat population contains resistance associated alleles at various frequency levels, some of which could be used for long term breeding programmes for a reduction of classical scrapie cases.

## Competing interests

The authors declare that they have no competing interests.

## Authors' contributions

WG conceived of the study, and participated in its design and coordination, performed the statistical analysis and drafted the manuscript. The molecular genetics study was carried out by KR, PS, DP, GS for the UK goats and by PS, RX, NF for the Mexican goats. OW, AB, JF participated in the design of the study. LG contributed to the design and the performance of the scrapie diagnosis and pathology study. All authors read and approved the final manuscript.
